# Management of Skin Pigmentation by a Combination of Alma Hybrid Laser and Transepidermally Delivered Phloretin: A Case Series

**DOI:** 10.1111/jocd.71284

**Published:** 2026-11-13

**Authors:** Greg Goodman, Tania Romana

**Affiliations:** ^1^ Monash University Clayton Victoria Australia; ^2^ University College London London UK; ^3^ Department of Microbiology, Anatomy, Physiology and Pharmacology La Trobe University Bundoora Victoria Australia; ^4^ SkinCeuticals Melbourne Victoria Australia

**Keywords:** Alma hybrid laser, antioxidant, hyperpigmentation, phloretin, skin, sonophoresis

## Abstract

**Background:**

The Alma Hybrid laser is a multi‐technology device that combines both ablative and non‐ablative lasers to target water and induce photo‐thermolysis for skin treatment, in a fractional manner. The combination of fractionation and sonophoresis enables a novel technological approach with the potential to significantly enhance the delivery and absorption of cosmeceuticals, such as phloretin, into and through the epidermis and into the dermis. Phloretin exhibits potent antioxidant properties that effectively neutralize free radicals and inhibit lipid peroxidation, helping to reduce inflammation and consequent facial discoloration.

**Aims:**

To present practical insights on the safety of transepidermal delivery of Phloretin CF serum for hyperpigmentation.

**Methods:**

Three case vignettes were selected to demonstrate the typical safety profile of this intervention.

**Results:**

Pain and stinging at the end of the laser treatment were mostly mild or moderate in intensity; subsequent application of phloretin induced extra symptomatology that was transient and resolved immediately following sonophoresis. Improvement in hyperpigmentation was observed, as indicated by both VISIA absolute scores and VISIA feature counts, as well as patient‐reported improvement in radiancy.

**Conclusions:**

This case series supports the safety and tolerability of delivering transepidermal Phloretin CF serum using fractional ablative CO_2_ laser combined with sonophoresis, whilst allowing for deeper serum penetration.

## 1 Introduction

Hyperpigmentation of the skin is a common dermatological condition characterized by the skin becoming darker, usually in an uneven way. Although hyperpigmentation is neither harmful nor life‐threatening, it can profoundly affect people's emotional and psychological wellbeing [1]. Current methods for treating hyperpigmentation include topical agents and laser therapy, both of which have limitations.

Topical agents are limited by adverse effects, require prolonged use, and have poor patient compliance and low satisfaction [2]. Furthermore, they have low transdermal absorption of active ingredients due to the skin barrier and molecular constraints [3].

Laser therapies, particularly ablative types such as fractional CO_2_ lasers, are effective for hyperpigmentation and have advanced the treatment landscape [4]. However, laser therapies are often associated with side effects such as erythema and postinflammatory hyperpigmentation (PIH) [5, 6]. Although PIH is generally benign and may resolve over time, it can significantly impact self‐esteem and overall quality of life [5, 6].

Laser procedures produce controlled micro‐injuries in the skin, which generate a large amount of reactive oxygen species that can delay recovery [7]. Antioxidants have been shown to be effective in reducing the adverse effects of ablative laser treatment by reducing the oxidative stress commonly caused [8, 9].

Phloretin, a phenolic compound derived from apples, exhibits potent antioxidant properties that effectively neutralize free radicals and inhibit lipid peroxidation [10, 11, 12]. It has been shown to inhibit matrix metalloproteinase‐1 (MMP‐1) and elastase enzymes, compounds that degrade connective tissue and play an important role in photoaging [12]. Phloretin has also been shown to inhibit tyrosinase activity in human melanocytes, indicating potential as a topical lightening agent for pigment disorders of skin [12]. Studies have also shown that phloretin can help reduce facial discolouration, indicating potential as a cosmetic agent for treating hyperpigmentation [10, 11].

While topically applied antioxidants can inhibit oxidative damage to the skin, the stratum corneum—the primary barrier—limits compound delivery through the epidermis. To enhance dermal interaction and activity, an improved method to enhance absorption of antioxidants is desirable [13]. To enhance compound delivery, some laser fractional ablation laser technologies are combined with sonophoresis of low‐frequency ultrasound waves. The combination of fractionation and sonophoresis allows a novel technological approach with the potential to enhance delivery and absorption of substances such as cosmeceuticals across the dermal layer with significantly better delivery [14]. Safety and healing issues limit older fully ablative technologies in achieving depths of dermal penetration that the fractional lasers are capable of delivering. Fractional ablative lasers, with their vertical columns of ablated tissue, enable safe bypass of the stratum corneum to reach the dermis and deeper levels of the epidermis [15, 16], while ultrasound sonophoresis temporarily increases skin barrier permeability due to its cavitation effects [2, 17]. Sonophoresis facilitates the penetration of topical agents into the laser‐induced microchannels by a push‐pull effect on applied substances after laser resurfacing, as well as this skin permeability change.

The Alma Hybrid laser is a multi‐technology combination device that uses both ablative (CO2) and non‐ablative (Erbium Glass 1570 nm) lasers to target water and induce photo‐thermolysis to treat the skin. It does so in a fractional manner, using many different patterns to achieve its effects.

Using a “paintbrush mode”, which places dots of treatment at random (but never juxtaposing sequential pulses next to each other), allowing for a very even and uniform resurfacing. This may be used at low power to just ablate the epidermis or at higher energies to insert deeper holes. This latter effect will allow epidermal effects with removal of pigmented spots and sun damage pigmentation while also allowing deeper penetration to help remodel the dermis. In cases of sun damage, both layers often require treatment. This controlled disruption of the skin barrier can also aid enhanced penetration of cosmetics, as the microchannels created by the laser facilitate deeper delivery of these topical agents.

While microchannels may enhance permeability, important factors to consider include the depth of ablation, the molecular size of the cosmetic ingredients, and the potential for irritation [18].

We conducted a study to determine whether the Alma Hybrid laser could facilitate phloretin delivery, and to assess its safety and tolerability in transepidermal drug delivery. It is thought that the enhanced penetration of phloretin through the skin could potentially maximize its potency. Written informed consent was obtained from the individuals who participated in the study. Patients were treated with Alma Hybrid Fractional Ablative CO_2_ laser, with (*n* = 17 patients) or without (*n* = 13 patients) phloretin serum. We found that the phloretin group showed no greater improvement in facial pigmentation than the Alma Hybrid laser alone group (data not shown), likely due to the effectiveness of the laser on its own, resulting in a high baseline effect such that the effect of topical phloretin was not detected within this cohort. Due to this effect, the primary endpoint was underpowered thus preventing efficacy analysis. However, valuable practical insights on the safety of using transepidermal delivery of phloretin were gained. In this wider group no safety concerns related to phloretin use were seen. Here we present three case vignettes from this study which document the typical safety profile. To objectively measure results, we used the VISIA Complexion Analysis System. The VISIA Complexion Analysis System is a widely used tool for a full facial skin analysis [19]. VISIA produces many measurements and the three different measurements, the percentile, the feature count, and the absolute score were used in this study [20]. The absolute score offers a comprehensive measure of the impact each feature has on the patient's complexion (on a scale from 0 to 100 for every skin characteristic, with lower scores indicating better outcomes). The feature count quantifies the number of discrete instances of the evaluated feature, regardless of the size or intensity of each instance (a reduction in the number of discrete instances of a particular feature is desired, with lower feature count numbers indicating an improvement). We measured the absolute score—generated by measuring brown spots/pigment, and the feature count (the sum of the individual areas of hyperpigmentation).

## 2 Case Series

All three patients received Alma Hybrid CO_2_ laser treatment for facial hyperpigmentation, using the paintbrush mode (30 watts, 1.2 milliseconds, 300 to 400 joules total energy per cosmetic unit). The cosmetic units were left and right cheeks, forehead and the combination of nose, upper lip, and chin together with phloretin serum (2% phloretin, 10% pure L‐ascorbic acid, and 0.5% ferulic acid [Phloretin CF, SkinCeuticals Inc.]). Sonophoresis was performed at a frequency of 50 Hz for a duration of 120 s with impact set at 30%. Patient characteristics are shown in Table 1. Laser treatments were administered at baseline, at Week 6, and at Week 12; phloretin serum was applied daily from baseline for up to 20 weeks.

**TABLE 1:** Patient characteristics, and treatment tolerability and pain outcomes.

Case	Sex	Age (years)	FST	Skin concerns	Key safety findings
C1	F	51	1	Global facial freckles, increased pigmentation, solar keratosis, significant facial sun damage	Temporary stinging and burning from lasers resolved with sonophoresis. Temporary (less than one minute) extra stinging and burning from phloretin use resolved spontaneously before sonophoresis
2	F	42	1	Enlarged pores, solar lentigines, global fine lines	Temporary stinging and burning from lasers resolved with sonophoresis. Temporary (less than one minute) extra stinging and burning from phloretin use resolved spontaneously before sonophoresis
3	F	60	3	Solar lentigines, flat seborrhoeic keratoses, ephelides	Temporary stinging and burning from lasers resolved with sonophoresis. Temporary (less than one minute) extra stinging and burning from phloretin use resolved spontaneously before sonophoresis

Abbreviation: FST, Fitzpatrick skin type.

### 2.1 Case 1

A 51‐year‐old female, with Fitzpatrick skin Type 1, presented with global facial freckles, increased pigmentation, solar keratosis and significant facial sun damage, likely resulting from years of inconsistent sun protection and lack of sunscreen use. Facial seborrhoeic dermatitis was observed, along with a reported lack of regular skincare. In a FACE‐Q baseline assessment, the patient reported dissatisfaction with nearly all aspects of her skin appearance and quality (Table 2). Her medical history included poorly differentiated squamous cell carcinoma on the ear, which was subsequently treated with Mohs surgery.

**TABLE 2:** VISIA and DLQI scores before treatment with Alma Hybrid laser and phloretin and at 20 weeks posttreatment.

Case	Baseline			Before treatment 1 day 1			Before treatment 2 week 6			Before treatment 3 week 12			Follow up week 20		
	VISIA		Total DLQI	VISIA		Total DLQI	VISIA		Total DLQI	VISIA		Total DLQI	VISIA		Total DLQI
	AbScore	Feature count		AbScore	Feature count		AbScore	Feature count		AbScore	Feature count		AbScore	Feature count	
1	35.95		2	36.23	352.33	2	29.12	258.66	1	26.48	224	1	23.75	195.33	0
2	25.06	210.66	3	26.45	236.33	6	23.14	202.67	4	21.37	174	1	20.69	163.33	2
3	30.99	236.33	3	29.85	219.33	8	21.11	151.33	2	23.58	166.67	0	19.1	123.67	0

*Note:* VISIA assessments were performed using the VISIA (Canfield Scientific) skin analysis system.

Abbreviations: AbScore, absolute score; DLQI, dermatology life quality index.

Starting December 2024, three sessions of fractional ablative (Alma Hybrid) laser treatment were commenced, with each session scheduled 6 weeks apart. Improvements in the evenness of skin color, decrease in visible skin spots (lentigines and actinic keratoses) and texture were observed (Figure 1, Tables 2 and 3), as confirmed by decreases in both the VISIA absolute scores and VISIA feature count from baseline to Week 20 (Table 2). A decrease in the total DLQI was observed from baseline to Week 20 (Table 2). At Week 20, FACE‐Q assessments improved overall to mostly satisfied or very satisfied (Table 3).

**FIGURE 1:**
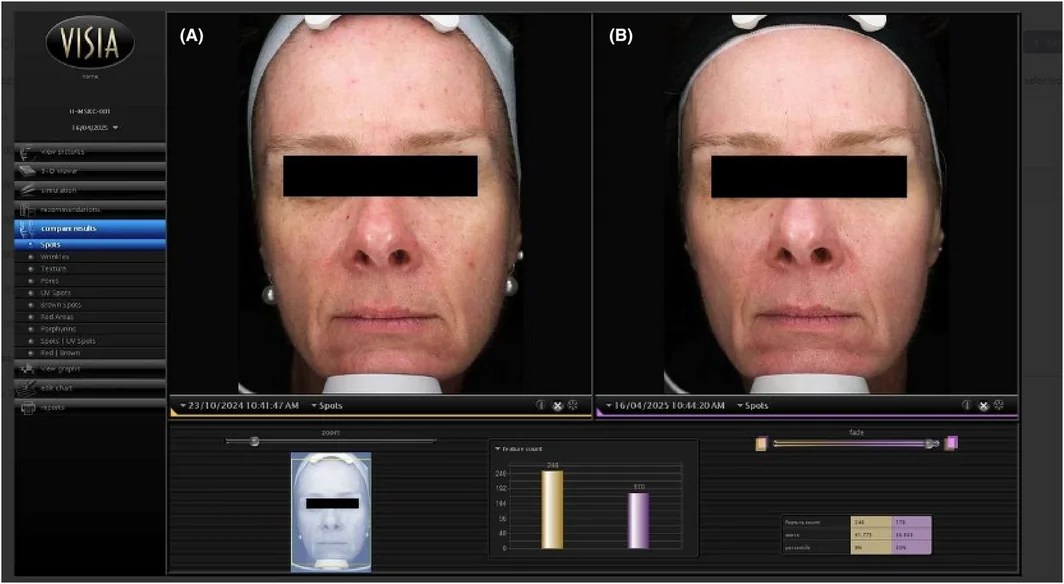
Case 1—VISIA at (A) baseline and (B) week 20 showing a marked decline in overall spots feature count from 351 to 195, raising the patient from the highest 5% spot count in her cohort to 35%.

**TABLE 3:** Patient‐reported FACE‐Q aesthetics scale assessment at baseline and 20 weeks posttreatment.

	Case 1		Case 2		Case 3	
	Baseline	Week 20	Baseline	Week 20	Baseline	Week 20
Q1. How your facial skin looks at the end of you day?	S	VS	D	D	D	S
Q2. How healthy your facial skin looks?	D	VS	D	D	D	S
Q3. How attractive your facial skin makes you look?	D	S	D	D	D	S
Q4. How smooth your facial skin looks?	D	VS	D	D	D	S
Q5. How clear your facial skin (complexion) looks?	D	VS	D	D	D	VS
Q6. How refreshed your facial skin makes you look?	D	VS	D	D	D	S
Q7. How hydrated your facial skin looks?	D	VS	D	D	D	S
Q8. How your facial skin looks when you first wake up?	D	VS	VD	D	D	S
Q9. How radiant your facial skin looks?	D	VS	VD	D	VD	S
Q10. How the tone (color) of your facial skin looks?	D	VS	VD	D	VD	S
Q11. How your pores look?	D	VS	D	D	D	S
Q12. How even‐colored your facial skin look?	D	VS	D	D	D	D

Abbreviations: D, dissatisfied; S, satisfied; VD, very dissatisfied; VS, very satisfied.

### 2.2 Case 2

A 42‐year‐old female, with Fitzpatrick skin Type 1, presented with enlarged pores, solar lentigines from sun damage, as well as global fine lines. The patient reported being very dissatisfied with the appearance of her face each morning, specifically noting a lack of radiance and an uneven skin tone in a FACE‐Q assessment (Table 3). Consistent use of a topical prescription retinoid (Retrieve) over several years did not effectively address her solar lentigines, sun damage, and overall pigmentation issues. The patient underwent laser treatment (Laser Genesis) in 2024.

In March 2025, the patient received the first of 3 sessions of fractional ablative laser treatment, each session scheduled 6 weeks apart, plus phloretin. Improvement in evenness of skin color and skin radiancy was observed (Figure 2, Tables 2 and 3), as confirmed by decreases in both the VISIA absolute scores and VISIA feature count from Day 1 (prior to first treatment) to Week 20 (Table 2). The total DLQI decreased from 6 on Day 1 to 2 on Week 20 (Table 2), although this was only partially reflected in the satisfaction outcome endpoint (Table 3). Breakouts, congestion, and comedones were observed after the first and second laser treatments, possibly from the hydrating balm used postoperatively requiring medical therapy (trifarotene). This may have impacted the patient's perception of improvement (Table 3).

**FIGURE 2:**
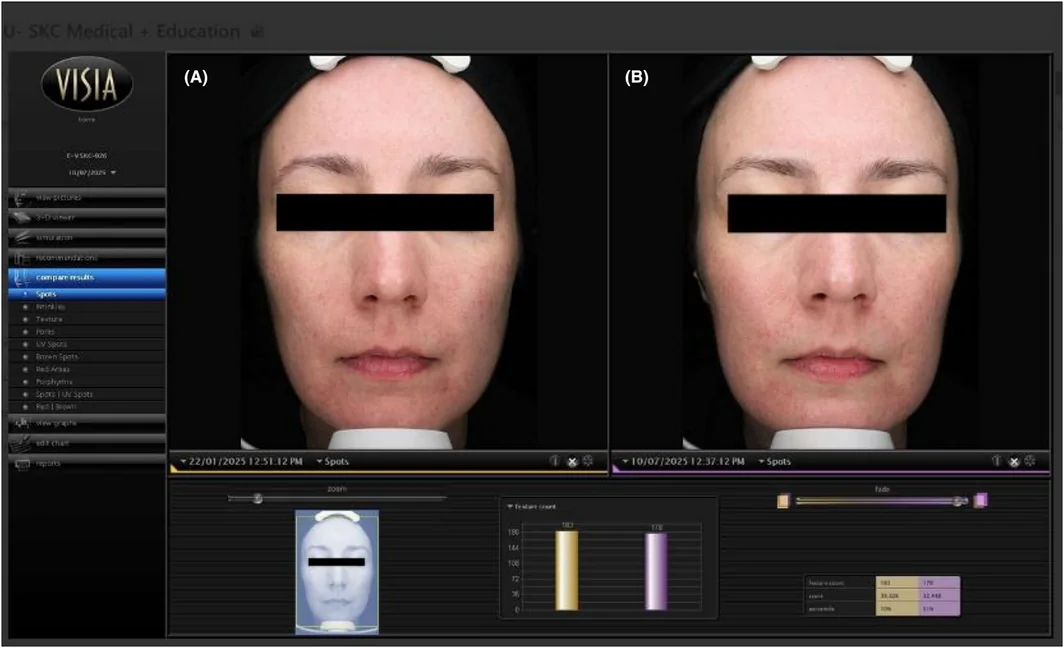
Case 2—VISIA at (A) baseline and (B) week 20 showing a modest change in spot count from 211 to 163.

### 2.3 Case 3

A 60‐year‐old postmenopausal woman, with Fitzpatrick skin Type 3, presented in April 2017 with concerns about solar lentigo‐like pigmentation on her left cheek, which began during pregnancy. Her history was notable for extensive sun exposure due to her occupation as a physical education teacher. In June 2021, she was diagnosed with widespread solar lentigines, flat seborrhoeic keratoses and ephelides in the photo distribution on her head, neck, chest and upper limbs. The patient participated in multiple treatment modalities for persistent hyperpigmentation. Liquid nitrogen, lasers (Excel V 532 nm and another fractional CO_2_), and intense pulsed light therapy were used to treat the background facial dyschromia.

In April 2025 the patient participated in this study and received fractional ablative laser treatment (Alma Hybrid). Following three cycles of Alma Hybrid laser treatments and phloretin serum applications the patient had a substantial decrease in the absolute number of spots and brown spots as well as pores (Figures 3 and 4). At follow up, the patient reported improved satisfaction on all FACE‐Q questionnaire metrics (Table 3) as well as improvement and healing of her solar lentigines. This corresponds with reductions in both the VISIA absolute score (from 30.99 to 19.1) and VISIA feature count (from 236.33 to 123.67) from baseline to Week 20 (Table 2). The total DLQI score decreased from 8 at Day 1 to 0 at Week 20 (Table 2).

**FIGURE 3:**
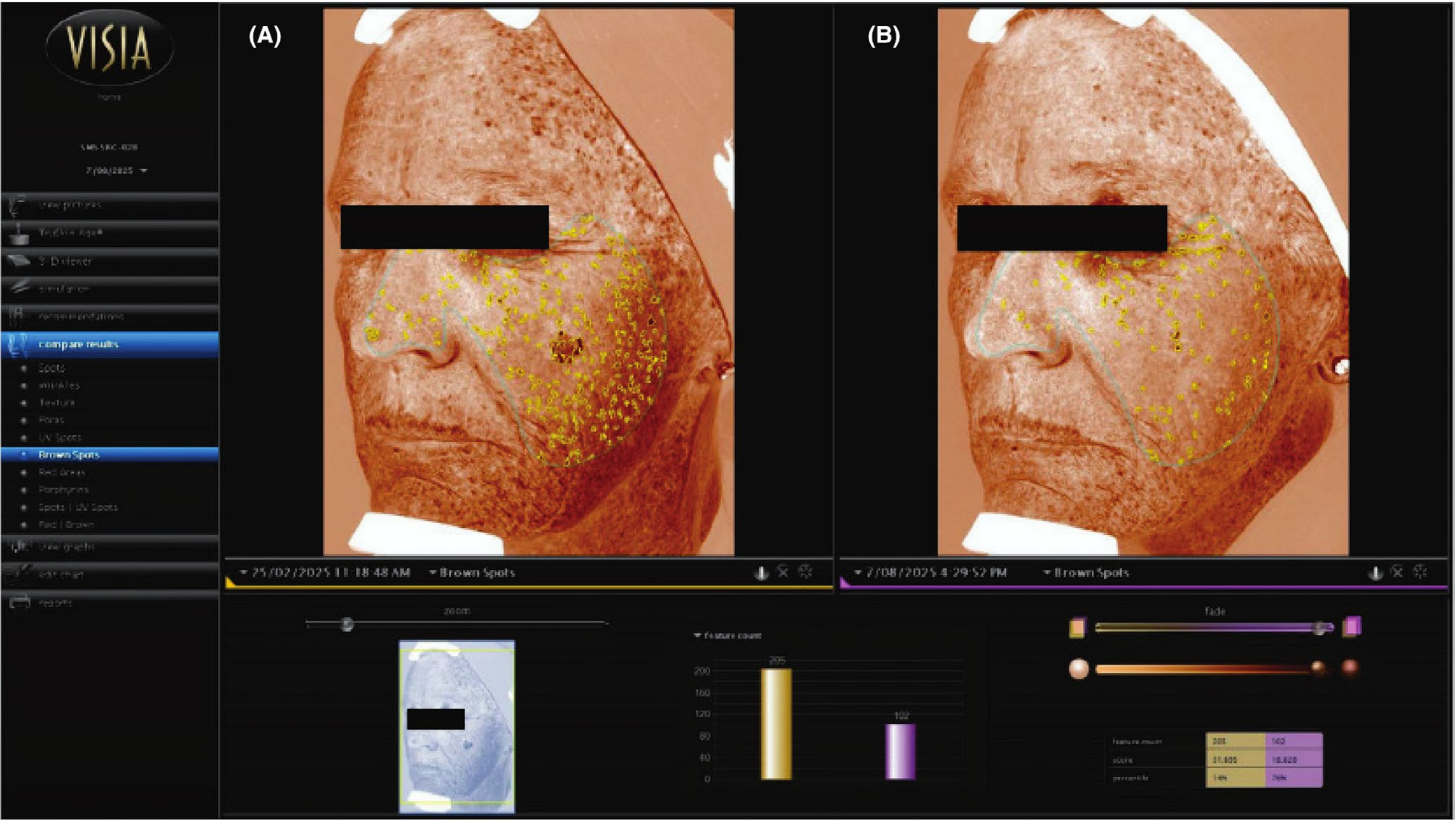
Case 3—VISIA brown spots (left view) at (A) baseline and (B) 6 months showing a decrease in brown spots on UV filter from 205 to 102 and changing her percentile in her cohort from 14% to 76%.

**FIGURE 4:**
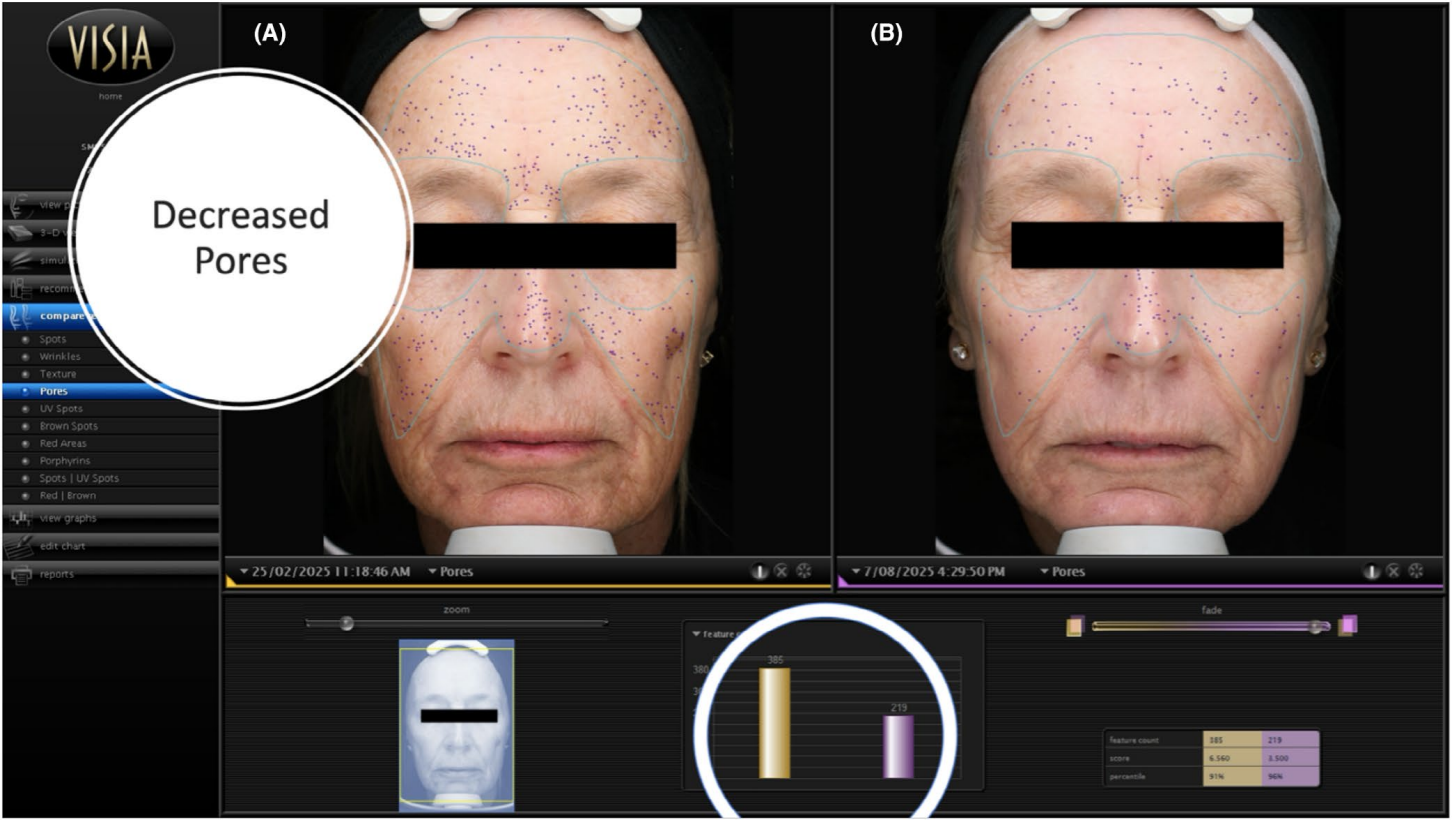
Case 3—VISIA—pores at (A) baseline and (B) 6 months. Although not a direct feature designed for assessment in this study, pores were automatically analyzed by the VISIA system and showed a decrease from 385 to 219.

In all patients, burning and stinging occurred after each session, with pain scores assessed and rated as 0 (none), 1 mild, 2 (moderate), or 3 (severe). Pain and stinging at the end of the laser treatment measured mostly mild or moderate. When applying phloretin, patients were asked whether the pain and stinging were increased by the phloretin application. These extra symptoms were mildly or moderately increased above the laser‐induced symptoms. However, this extra symptomatology from phloretin application was short‐lived, lasting less than one minute. After the subsequent sonophoresis, pain and stinging from the laser were completely and immediately settled. This was an observation seen in all sonophoresis cases.

## 3 Discussion

In our case series, transepidermal delivery of phloretin serum using Alma Hybrid laser and sonophoresis was well tolerated, with no serious safety issues observed. To our knowledge, sonophoresis has not been previously documented as a method to enhance topical phloretin delivery. Phloretin application via this method induced mild and transient stinging, lasting approximately 20 s, but no more than 1 min. This is in line with the typical irritation caused by L‐ascorbic acid and low pH formulations [21], rather than necessarily from the phenolic component of the phloretin. We also observed the unexpected finding that sonophoresis reduced the stinging sensations induced by both the laser and phloretin application. This may be attributed to vibrations produced by ultrasound waves and the gate control theory for pain—an established mechanism for coding the nociceptive component of cutaneous sensory input, although the complete reason for this significant and immediate pain relief is still ill understood [22].

Higher treatment densities in fractional laser resurfacing have been shown to increase the risk of PIH [6]. We used the paintbrush mode of the Alma Hybrid laser using the CO_2_ wavelength at 30 watts and 1.2 milliseconds. The paintbrush setting allowed the dispersion of energy (300 to 400 joules per facial area—each cheek, forehead and nose/perioral) evenly, with time for thermal relaxation, limiting heat build‐up between pulses as the scattered pulses are not delivered immediately next to each other during the treatment. We are not aware of previous publications on the paintbrush mode to deliver energy for laser assisted drug delivery. This setting has the potential to maintain patient comfort, perform an even resurfacing (with even dispersion of topically or sonophoresed agents) and minimize the risk of PIH. This modality was well tolerated, with no safety issues observed in this case series aside from the isolated acne breakout in Case 2.

Ablative fractional lasers, such as fractional CO_2_, are associated with a higher incidence of PIH compared with non‐ablative modalities [6] While patients with pigmentary concerns may be more prone to developing PIH, individuals with high Fitzpatrick skin phototypes carry a higher risk of PIH [5, 23, 24]. Whilst our cases were limited to Fitzpatrick photoageing skin Types 1–3, it was heartening that PIH was not observed in any case. Although Fitzpatrick skin Type 3 individuals are prone to pigmentation with CO2 lasers, the Alma Hybrid laser combined with phloretin did not exacerbate hyperpigmentation in our patient with this skin type (Figures 3 and 4).

Treatments such as fractional ablative CO_2_ are associated with reactivation of herpes simplex rates (1.77%; retrospective study of 961 successive treatments with fractional ablative CO_2_) [5]. In our case series, antiviral and antibiotic prophylaxis were not used, and reactivation of herpes simplex infections was not observed.

Overall, all three patients in our series demonstrated improvement in both VISIA absolute scores (Figure 5) and VISIA feature counts from Day 1 to Week 6, which continued progress was observed at Week 12 and Week 20 (Table 2).

**FIGURE 5:**
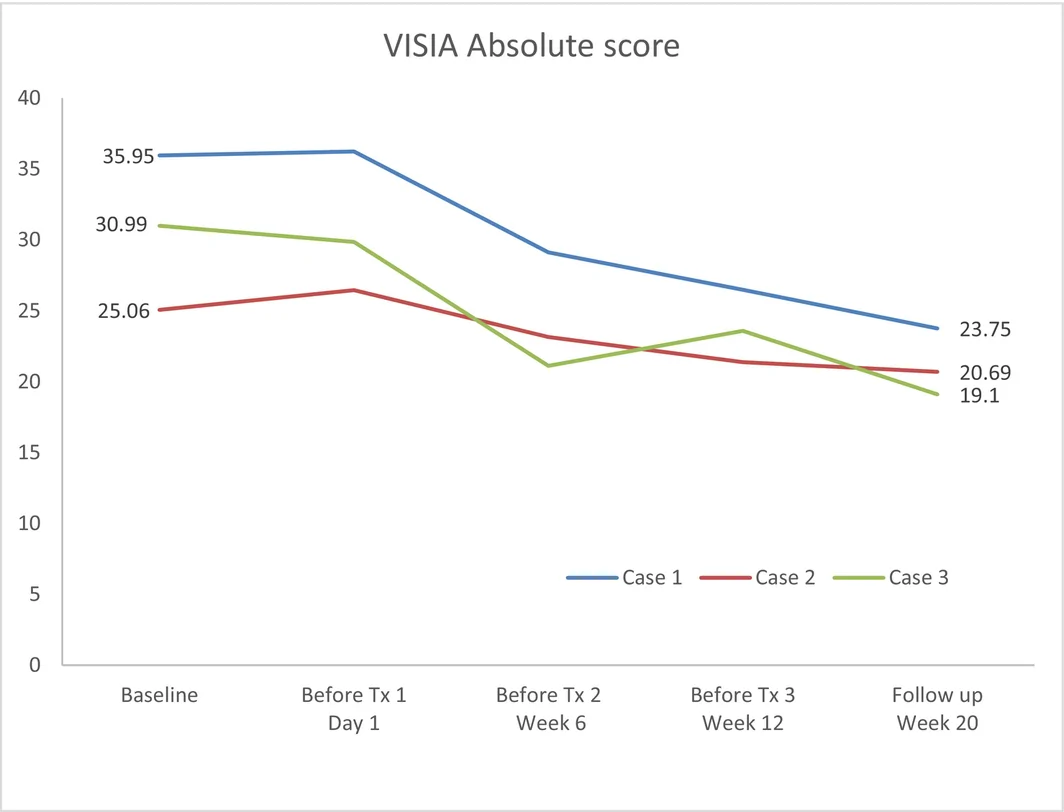
Absolute VISIA scores. All three patients showed a decline in absolute (feature count) scores with treatments. Tx, treatment.

The Dermatology Life Quality Index (DLQI) is a validated dermatology‐specific patient‐reported outcome tool to assess health‐related quality of life (QoL) [25, 26]. This 10‐item questionnaire explores the impact of skin diseases on daily activities and social interactions, with responses rated on a scale from 0 (absent) to 3 (severe). Individual scores are cumulatively tallied to produce a total score ranging from 0 to 30, where higher scores indicate greater QoL impairment. In general populations, the mean DLQI scores (maximum 30) range from 0 to 0.5 [25]. A change of 4 points is widely accepted as the minimal clinically important difference for the DLQI in inflammatory skin diseases [27]. An increase in DLQI was observed from baseline to Day 1 (both pre‐treatment) in Cases 2 and 3; although the reason for this is unclear, patients viewed their VISIA scores between these two assessments, which may have influenced their perceptions about the severity of their pigmentation and DLQI scores (Table 2). Our case series showed an overall downward trend on DLQI over time. DLQI scores for two patients recorded on Day 1 were 6 and 8, and decreased to 1 and 0, respectively by Week 20, highlighting the perceived impact of treatment from the patients' perspective.

Although this study was primarily involved in assessing pigmentation improvement after these treatments, patients also reported that their facial skin appeared smoother, their tone improved, and their pores were reduced (Table 3 and Figure 4). This suggests that their rejuvenation was more than just the coloring improvement. This is certainly suggested in Figure 4 where the patient's skin wrinkling in the lower face appears significantly better.

Radiancy is an interesting outcome (Table 3) and is a manifestation of both epidermal and dermal issues. Evening out the color and removal of excess pigmentation is one aspect but dermal correction and improvement are also important in allowing the glow or radiancy to become more obvious. All patients noted an improvement in this factor after the treatments (Table 3).

## 4 Conclusions

This case series supports the safety and tolerability of delivering transepidermal Phloretin CF serum using fractional ablative CO_2_ laser combined with sonophoresis to enhance phloretin absorption. It shows that phloretin is well tolerated and free of any major issues of delivering this agent deeply into the skin. Antioxidants are agents that are hard to deliver reliably to dermal tissues. This study acts as a guide to allow further exploration of delivery of such agents to stimulate deeper structures such as collagen and elastin fibers as well as successfully treating skin surface issues such as pigmentation.

This intervention has the potential to serve as an alternative to fractional ablative CO_2_ alone, offering enhanced rejuvenation through deeper penetration of serum. These findings are subject to the inherent limitations of a case series, and further research is needed to be conducted to verify these results.

## Author Contributions

The authors conceptualized and contributed to the design of the manuscript. The authors contributed to the development of the manuscript through critical review.

## Funding

Medical writing support was funded by SkinCeuticals, a L'Oreal company.

## Ethics Statement

This study was approved by the Bellberry Human Research Ethics Committee (approval no.: 2024–06‐762).

## Consent

Written informed consent was obtained from all individual participants prior to their inclusion in the study, and the research was conducted in accordance with the ethical principles of the Declaration of Helsinki. The participants in the study allowed the recording of their photographs to be used for the manuscript and its publication.

## Conflicts of Interest

Greg Goodman: Paid consultant for Allergan/Abbvie, Galderma, Caliway, Wesfarmers Health, L'Oreal, Inova. Tania Romano: Employee of SkinCeuticals, a L'Oreal company.

## Data Availability

The data that support the findings of this study are available from the corresponding author upon reasonable request.
